# Mortality Rates from Cancer or All Causes and SOD Activity Level and Zn/Cu Ratio in Peripheral Blood: Population-based Follow-up Study

**DOI:** 10.2188/jea.12.14

**Published:** 2007-11-30

**Authors:** Yoshinori Ito, Koji Suzuki, Ryuichiro Sasaki, Motohiko Otani, Kunio Aoki

**Affiliations:** 1 Department of Public Health, Fujita Health University School of Health Sciences, Toyoake, Japan.; 2 Nagano Prefectural Saku Public Health Station, Saku, Japan.; 3 Department of Hygiene, Fujita Health University School of Medicine, Toyoake, Japan.; 4 Aichi Cancer Center, Nagoya, Japan.

**Keywords:** SOD activity, TBARS, cancer death, follow-up, zinc

## Abstract

A total of 507 residents (232 males and 275 females) of a rural area of Hokkaido, Japan, were enrolled in the present follow-up study as the follow-up cohort. We investigated the relationship between mortality rates from cancer or all causes and blood levels of SOD activity and Zn/Cu ratio, and serum levels of SOD activity and lipid peroxides (TBARS), by following up our subjects over the course of 18 years. The hazard ratios of lowest blood levels of SOD activity and Zn/Cu ratios to mortality rates from cancer were 1.36 (95% C.I., 0.59-3.10) and 1.97 (95% C.I., 0.84-4.63), and those to mortality rates from all causes were 1.31 (95%C.I: 0.76-2.27) and 1.99 (95%C.I.: 1.14-3.46), respectively, in comparison with subjects with highest values for these factors. Hazard ratios of lowest serum levels of SOD activity and TBARS to mortality rates from cancer were 2.68 (95%C.I., 1.05-6.82) and 0.71 (95%C.I.: 0.31-1.67), and those to mortality rates from all causes were 1.55 (95% C.I., 0.90-2.66) and 0.88 (95%C.I.: 0.51-1.50), respectively. The results of this follow-up study indicate that high serum SOD activity is associated with protective effects against mortality from cancer, and that high blood Zn/Cu ratio is associated with low mortality from other causes. In contrast, serum TBARS levels do not appear to be a biomarker for the risk of death from cancer.

## INTRODUCTION

Superoxide dismutase (SOD) is an enzyme that plays an important role in biological defense against carcinogenesis caused by activated oxygen and/or free radicals by reducing formation of activated oxygen from superoxide anions^1^^-^^3^^)^. SOD activity is well known to be affected by aging and some diseases^3^^-^^7^^)^, as well as blood and tissue levels of activated oxygen and free radicals^7^^-^^10^^)^. There are 3 isozymes of mammalian SOD: Cu, Zn-SOD, extracellular SOD and Mn-SOD, which are located in cytosol, extracellular fluid and mitochondrial matrix, respectively^11^^)^. Cu, Zn-SOD is present in the cytosol of various tissues (including liver, cerebral gray matter, cardiac muscle and testes), and serum Cu, Zn-SOD is derived mainly from cytosol and extracellular fluid of these tissues, although this enzyme is also released from erythrocytes by hemolysis^11^^)^. Recently, it was reported that blood activity of SOD, including SOD in erythrocytes, appears to be lower among patients with cancer of the lung, esophagus, stomach, colorectum or pancreas^12^^,^^13^^)^. Although SOD activity in erythrocytes is elevated in patients with hematologic neoplasms^14^^)^, SOD activity in erythrocytes decreases with increasing duration of treatment for leukemia and various visceral cancers^15^^)^. Serum SOD activity in patients with leukemia or non-Hodgkins lymphoma is significantly lower than normal^16^^)^. It has been reported that serum Cu, Zn- and Mn-SOD activities in serum and pleural fluids are higher in patients with squamous cell carcinoma of the lung or tuberculosis than in controls^17^^)^, whereas serum Cu, Zn-SOD activity levels are reportedly lower in malignant tissues of stomach mucosa than in normal tissues^18^^)^. However, there have been scarcely any reports of population-based follow-up studies demonstrating that cancer death is related to blood SOD activity level.

Some trace metals are essential elements which function as cofactors of various enzymes or have other biological functions [e.g., zinc (Zn) and copper (Cu) are cofactors of SOD], although some metals can be toxic at excessively high levels^19^^-^^22^^)^. It has been reported that Zn levels in plasma, erythrocytes and whole blood are lower in cancer patients^23^^)^. In malignant tissues, Zn levels are reportedly low, whereas copper levels are reportedly high^24^^)^. Similarly, in serum from cancer patients, levels of zinc are reportedly low and levels of copper are reportedly high^25^^,^^26^^)^.

In this study, we investigated whether subjects with high levels of SOD activity in their serum or blood and high Zn/Cu ratios in their blood have a low risk of mortality from cancer or all causes.

## SUBJECTS AND METHODS

### SUBJECTS

The subjects consisted of a population cohort of 507 healthy residents (232 males and 275 females) of a rural area of Hokkaido, Japan, who attended a health check in August, 1982. All participants were over 40 years of age, and the majority of them worked in dairy farming, fishing or commerce.

Medical examinations for these subjects continued for 20 years following the initial health check. Public health nurses interviewed individuals and administered questionnaires on health and lifestyle at the time of the health check. Lifestyle habits queried included smoking (current smoker, ex-smoker, never smoked), alcohol consumption (regular drinker, occasional drinker, non-drinker) and dietary intake of major food groups^27^^)^. Prior history of disease, dietary habits and other lifestyle factors did not differ significantly between the present cohort and other residents of their town^27^^)^. Subjects (residents of Y-town) were not randomly selected, but their residences were scattered across their town relatively evenly (not in clusters), and their sex and age distributions were similar to the overall distributions of their town. There were no significant differences in history of previous diseases, diet or lifestyle between our cohort and the overall population of their town.

The cohort was followed up until the end of December, 1999, using mortality records at the Public Health Center to confirm whether subjects had survived for the entire follow-up period or had died. Permission to use these records was obtained from the Agency of General Affairs and the Ministry of Health and Welfare. Death certificates were examined to determine the main cause of death and contributing factors. Only 18 of the cohort subjects changed residence during the observation period. Because the number of deaths was reliably reported, we calculated mortality rates using the person-year method.

### METHODS

Fasting blood samples were taken at the time of the health check, and the sera were separated from blood cells by centrifugation within one hour. Biochemical analysis of the sera was performed using an autoanalyser. Serum TBARS was fluorometrically measured by the thiobarbituric acid-reaction method^28^^)^. Blood and serum SOD activity was determined using superoxide anions produced by NADPH cytochrome c reductase in rat microsomes as the substrate and neo-tetrazolium salt as the color reagent^29^^)^, after removal of hemoglobin^30^^)^. Serum and blood SOD activity levels and serum TBARS levels were estimated using an established method within 2 months of sample collection. Blood samples were collected into metal-free tubes at the time of the health examinations and stored in a freezer at -80°C. Blood levels of Zn and Cu were determined by anodic stripping voltammetry^31^^)^ after burning the blood samples to ash with a mixed acid solution (HNO_3_ :H_2_ SO_4_ :HClO_4_ = 24:1:4, v/v), within 2 months of sample collection. We could not determine serum levels of Zn and Cu, because of inadequate sample volume and/or failure to ensure that the sample storage cup was metal-free.

### STATISTICAL ANALYSIS

Statistical analyses for each age group were carried out using a Cox proportional hazard model (statistical package: StatView 5.0, Power Macintosh), after adjusting for sex and age (Model 1). Statistical analyses for Model 2 were performed after adjusting for sex, age, BMI, smoking status and alcohol consumption. Statistical significance was set at p<0.05. The ANOVA was conducted using the StatView 5.0 statistical package, after adjusting for sex and age. Deaths from all causes were separated into 2 groups: deaths from all causes and deaths from all causes other than accidents or cancer of all sites. The hazard ratios (H.R.) and 95% confidence intervals (95% C.I.) were calculated for groups of subjects classified according to their quartile distribution for levels of the assayed blood factors: Q1 (low), within the first quartile; Q2, in the second quartile; Q3, in the third quartile; Q4 (high), in the fourth quartile.

## RESULTS

Table 1 shows the subject characteristics of the present study. The subjects were separated into 2 groups: below 60 years of age, and 60 years of age or older. The group of subjects 60 years of age or older had a smaller percentage of females. Current smokers and regular alcohol drinkers accounted for 52.6% and 66.0% of all males, respectively, and 5.5% and 11.3% of all females. During the 18-year follow-up period, 106 deaths (73 males and 33 females) occurred among the cohort. The breakdown of the deaths was as follows: 40 from malignant neoplasm (30 males and 10 females); 13 from ischemic heart diseases (6 males and 7 females); 11 from cerebrovascular diseases (7 males and 4 females); 8 from accidents (6 males and 2 females); and 29 from other causes (19 males and 10 females). No deaths occurred during the first year of the follow-up. Eighteen subjects changed residence (5 males and 13 females) during the 18-year follow-up period.

**Table 1.** Baseline characteristics of the follow-up subjects.

Item		Total (%)		Males (%)						Females (%)					
	
				Age group						Age group					
				40-59		60-86		Overall (%)		40-59		60-86		Overall (%)	
Subjects		507	(100.0)	128	(100.0)	104	(100.0)	232	(100.0)	188	(100.0)	87	(100.0)	275	(100.0)

Habits	Current smoker	137	( 27.0)	75	( 60.0)	47	( 45.2)	122	( 52.6)	7	( 3.7)	8	( 9.2)	15	( 5.5)
	Alcohol drinker	184	( 36.3)	83	( 66.4)	70	( 67.3)	153	( 66.0)	15	( 8.0)	22	( 25.3)	31	( 11.3)

Death	All causes	106	(100.0)	22	(100.0)	51	(100.0)	73	(100.0)	11	(100.0)	22	(100.0)	33	(100.0)

	Cancer of all sites	40	( 37.7)	11	( 50.0)	19	( 37.3)	30	( 41.1)	7	( 63.6)	3	( 13.6)	10	( 30.3)
	Circulatory disease	29	( 27.4)	4	( 18.2)	14	( 27.5)	18	( 24.7)	1	( 9.1)	10	( 45.5)	11	( 33.3)
	Ishemic heart disease	13	( 12.3)	1	( 4.5)	5	( 9.8)	6	( 8.2)	0	( 0.0)	7	( 31.8)	7	( 21.2)
	Cerebrovascular disease	11	( 10.4)	1	( 4.5)	6	( 11.8)	7	( 9.6)	1	( 9.1)	3	( 13.6)	4	( 12.1)
	Accidents	8	( 7.5)	4	( 18.2)	2	( 3.9)	6	( 8.2)	2	( 18.2)	0	( 0.0)	2	( 6.1)
	Others	29	( 27.4)	3	( 13.6)	16	( 31.4)	19	( 26.0)	1	( 9.1)	9	( 40.9)	10	( 30.3)

Serum SOD activity was lower for subjects who died of cancer, but there were no significant differences between subjects who died and those who remained alive (Table 2). Serum TBARS levels tended to be higher for subjects who died than for those who remained alive. There were no clear differences in levels of blood SOD activity or Zn/Cu ratios between subjects who died and those who remained alive, although blood levels of Zn and Cu could not be determined for one of the subjects who died because of inadequate sample volume. Among deaths from all causes other than accidents and deaths from cancer of all sites, there were similar levels of blood SOD activity and similar Zn/Cu ratios between subjects who died and those who remained alive, whereas serum SOD activity appeared to be lower for subjects who died than for those who remained alive, but not significantly. In contrast, serum TBARS levels tended to be higher among subjects who died.

**Table 2.** Comparison of blood levels of SOD activity and Zn/Cu ratio, and serum levels of SOD activity and TBARS between the alive and the dead.

Component		Cancer of all sites					All causes							
	
		Alive		Dead		p	Alive		Dead (1)		p	Dead (2)		p
Blood SOD activity	(unit/ml)	462	(188)	475	(212)	0.68	462	(189)	4.64	(1.92)	0.92	470	(195)	0.69
Blood Zn/Cu ratio		7.83	(2.46)	8.02	(2.83)	0.64	7.84	(2.52)	7.83	(2.05)	0.98	7.85	(2.39)	0.98
SerumSOD activity	(unit/ml)	2.55	(1.13)	2.42	(1.13)	0.47	2.57	(1.18)	2.42	(0.86)	0.36	2.43	(0.94)	0.27
Serum TBARS	(nmol/ml)	5.09	(1.47)	5.31	(1.59)	0.35	5.09	(1.49)	5.12	(1.31)	0.87	5.17	(1.44)	0.61

Number		467		40			401		58			106		

Data represented are mean values and standard deviation in parenthesis.

Statistical analyses were conducted using ANOVA after adjusting for sex and age.

Dead (1): except for deaths from accidents and cancer of all sites.

Dead (2): total deaths.

Blood levels of SOD activity, blood Zn/Cu ratios and serum levels of SOD activity and TBARS were each categorized into 4 groups according to quartile distribution, from Q1 (low) to Q4 (high). Hazard ratios of blood levels of SOD activity, blood Zn/Cu ratios and serum levels of SOD activity and TBARS to cancer mortality rates calculated after adjusting for sex and age (Model 1) are shown in Table 3. The hazard ratios of blood SOD activity levels to cancer mortality were higher for the lowest-level group (Q1) than for the highest-level group (Q4). The hazard ratios of blood Zn/Cu ratios appeared to be higher for subjects with the lowest Zn/Cu ratios (Q1) than subjects with the highest ratios (Q4), but this difference was not statistically significant. The hazard ratios of serum SOD activity to cancer mortality were higher for subjects with the lowest (Q1) and middle (Q2 and Q3) activity levels than for subjects with the highest levels (Q4); the difference between the lowest-level group (Q1) and the highest-level group (Q4) was statistically significant. In contrast, hazard ratios of serum TBARS levels tended to be lower for subjects with lowest (Q1) and middle (Q3) serum TBARS levels, but this was not statistically significant.

**Table 3.** Hazard ratios of mortality for cancer of all sites by blood levels of SOD activity and Zn/Cu ratio, and serum levels of SOD activity and TBARS among the follow-up residents.

Components	Quartile	No. of subjects	Person years	No. of death	Mortality rates	Model 1	p	Model 2	p
						H.R.(95%C.I.)		H.R.(95%C.I.)	
Blood SOD activity (unit/ml)	Q1 (low)	129	2131.5	13	6.10	1.36 (0.59-3.10)	0.47	1.20 (0.51-2.80)	0.68
	Q2	135	2226.0	9	4.04	0.99 (0.40-2.43)	0.98	0.86 (0.35-2.16)	0.75
	Q3	115	1880.5	8	4.25	1.05 (0.41-2.67)	0.92	0.92 (0.36-2.37)	0.86
	Q4 (high)	128	2177.5	10	4.59	1.00		1.00	
						trend p=0.95		trend p=0.88	

Blood Zn/Cu ratio	Q1 (low)	126	2038.5	13	6.38	1.97 (0.83-4.63)	0.12	1.99 (0.84-4.69)	0.11
	Q2	129	2146.0	9	4.19	1.14 (0.45-2.88)	0.78	1.06 (0.41-2.69)	0.90
	Q3	125	2055.0	9	4.38	1.11 (0.44-2.79)	0.83	1.15 (0.45-2.91)	0.77
	Q4 (high)	126	2171.5	9	4.14	1.00		1.00	
						trend p=0.36		trend p=0.32	

Serum SOD activity (unit/ml)	Q1 (low)	156	2580.0	17	6.59	2.68 (1.05-6.82)*	0.04	3.16 (1.18-8.41)*	0.02
	Q2	115	1838.5	8	4.35	1.27 (0.44-3.68)	0.65	1.39 (0.48-4.04)	0.55
	Q3	116	1963.5	9	4.58	1.54 (0.55-4.33)	0.41	1.77 (0.62-5.05)	0.29
	Q4 (high)	120	2033.0	6	2.95	1.00		1.00	
						trend p=0.12		trend p=0.08	

Serum TBARS level (nmol/ml)	Q1 (low)	143	2411.5	10	4.15	0.71 (0.31-1.67)	0.43	0.80 (0.34-1.90)	0.62
	Q2	118	1951.0	11	5.64	1.00 (0.45-2.24)	0.99	1.14 (0.50-2.60)	0.76
	Q3	130	2145.0	6	2.80	0.43 (0.16-1.13)	0.09	0.46 (0.17-1.22)	0.12
	Q4 (high)	116	1908.0	13	6.81	1.00		1.00	
						trend p=0.30		trend p=0.31	

Hazard ratios (H.R.) and 95% confidence intervals (95%C.I.) were calculated using Cox proportional hazard model(StatView statistical package, Power Macintosh), after adjusting for sex and age (Model 1), or after adjusting for sex, age, habit of smoking status and alcohol consumption, and BMI (Model 2).

* : p<0.05.

Mortality rates = [No. of deaths/person years] × 1000

After adjusting for sex, age, BMI, smoking status and alcohol consumption (Model 2), the hazard ratios of blood or serum levels of SOD activity to cancer mortality were higher for subjects with the lowest activity levels (Q1) than the subjects with the highest activity levels (Q4); for serum SOD activity, the difference was statistically significant. Also, the hazard ratios of blood Zn/Cu ratio were higher for subjects with the lowest ratio (Q1). In contrast, hazard ratios of serum TBARS levels were lower for subjects with the lowest (Q1) and middle (Q3) levels, but this was not statistically significant.

Among deaths for all causes, except from deaths of accidents or cancer of all sites, hazard ratios of blood SOD activity were higher for subjects with low and middle activity levels than for those with the highest activity levels (Q4) (Table 4). The hazard ratios of blood Zn/Cu ratios were also higher for subjects with low and middle Zn/Cu ratios, but these differences were not statistically significant. The hazard ratios of serum SOD activity appeared to be higher for subjects with the lowest (Q1) and middle (Q2) activity levels, similar to the pattern seen above with hazard ratios of blood Zn/Cu ratio. There was a statistically significant difference in hazard ratios of serum SOD activity levels between subjects with middle activity levels (Q2) and those with the highest activity levels (Q4). We found that subjects with the lowest (Q1) serum TBARS levels tended to have slightly lower hazard ratios to mortality from cancer of all sites.

**Table 4.** Hazard ratios of mortality for all causes, except for accidents and cancer of all sites by blood levels of SOD activity and Zn/Cu ratio, and serum levels of SOD activity and TBARS among the follow-up residents.

Components	Quartile	No. of subjects	Person years	No. of death	Mortality rates	Model 1	p	Model 2	p
						H.R.(95%C.I.)		H.R.(95%C.I.)	
Blood SOD activity (unit/ml)	Q1 (low)	115	2010.0	15	7.46	1.20 (0.56-2.58)	0.64	1.35 (0.64-2.97)	0.46
	Q2	123	2092.5	20	9.56	1.80 (0.87-3.70)	0.11	1.61 (0.77-3.35)	0.21
	Q3	104	1812.5	11	6.07	1.48 (0.64-3.39)	0.36	1.48 (0.63-3.45)	0.37
	Q4 (high)	117	2052.0	12	5.85	1.00		1.00	
						trend p=0.40		trend p=0.63	

Blood Zn/Cu ratio	Q1 (low)	108	1874.0	12	6.40	1.99 (0.87-4.56)	0.10	1.95 (0.85-4.48)	0.12
	Q2	120	2056.5	18	8.75	1.98 (0.93-4.26)	0.08	2.23 (1.03-4.79)*	0.04
	Q3	116	1987.0	17	8.56	1.71 (0.80-3.67)	0.17	1.94 (0.90-4.21)	0.09
	Q4 (high)	115	2049.5	11	5.37	1.00		1.00	
						trend p=0.28		trend p=0.20	

Serum SOD activity (unit/ml)	Q1 (low)	139	2429.5	17	7.00	1.67 (0.80-3.49)	0.14	1.64 (0.76-3.55)	0.21
	Q2	103	1738.0	19	10.93	2.15 (1.04-4.42)*	0.04	2.07 (0.99-4.31)	0.05
	Q3	106	1857.0	9	4.85	1.04 (0.44-2.48)	0.93	0.84 (0.36-2.14)	0.78
	Q4 (high)	111	1942.5	13	6.69	1.00		1.00	
						trend p=0.12		trend p=0.09	

Serum TBARS level (nmol/ml)	Q1 (low)	130	2274.0	13	5.72	0.88 (0.41-1.92)	0.76	0.74 (0.33-1.67)	0.47
	Q2	106	1854.5	12	6.47	1.19 (0.54-2.64)	0.66	1.03 (0.45-2.34)	0.94
	Q3	123	2086.5	20	9.59	1.11 (0.55-2.25)	0.77	1.18 (0.58-2.42)	0.65
	Q4 (high)	100	1752.0	13	7.42	1.00		1.00	
						trend p=0.88		trend p=0.65	

Hazard ratios (H.R.) and 95% confidence intervals (95%C.I.) were calculated using Cox proportional hazard model (StatView statistical package, Power Macintosh), after adjusting for sex and age (Model 1), or after adjusting for sex, age, habits of smoking status and alcohol consumption, and BMI (Model 2).

* : p < 0.05.

Mortality rates = [No. of deaths/ person years] × 1000

Similar results were obtained after adjusting for sex, age, BMI, smoking status and alcohol consumption (Model 2). However, compared to Model 1, there was less of a difference in hazard ratios of serum SOD activity between the lowest levels (Q1) and the highest levels (Q4). There was a statistically significant difference in hazard ratios of blood Zn/Cu ratio between Q2 levels and Q4 levels.

Among deaths from all causes, hazard ratios of blood SOD activity were higher for subjects with low and middle activity levels than for those with the highest activity levels (Q4) (Table 5). The hazard ratios of blood Zn/Cu ratios were also higher for subjects with low and middle Zn/Cu ratios, and the differences between subjects with the lowest levels (Q1) and those with the highest levels (Q4) were statistically significant. The hazard ratios of serum SOD activity to mortality from all causes appeared to be higher for subjects with the lowest (Q1) and middle (Q2) activity levels; the differences were not significant. We found that subjects with the lowest (Q1) serum TBARS levels tended to have slightly lower hazard ratios to mortality from all causes.

**Table 5.** Hazard ratios of mortality from all causes by blood levels of SOD activity and Zn/Cu ratio, and serum levels of SOD activity and TBARS among the follow-up residents.

Components	Quartile	No. of subjects	Person years	No. of death	Mortality rates	Model 1	p	Model 2	p
						H.R.(95%C.I.)		H.R.(95%C.I.)	
Blood SOD activity (unit/ml)	Q1 (low)	129	2131.5	29	13.61	1.31 (0.76-2.27)	0.33	1.35 (0.77-2.37)	0.29
	Q2	135	2226.0	32	14.38	1.55 (0.90-2.65)	0.11	1.45 (0.84-2.50)	0.18
	Q3	115	1880.5	22	11.70	1.36 (0.76-2.45)	0.30	1.26 (0.69-2.31)	0.45
	Q4 (high)	128	2177.5	23	10.56	1.00		1.00	
						trend p=0.46		trend p=0.57	

Blood Zn/Cu ratio	Q1 (low)	126	2038.5	30	14.72	1.99 (1.14-3.46)*	0.02	2.02 (1.16-3.52)*	0.01
	Q2	129	2146.0	27	12.58	1.49 (0.85-2.62)	0.17	1.50 (0.85-2.66)	0.16
	Q3	125	2055.0	25	12.17	1.35 (0.76-2.38)	0.30	1.40 (0.79-2.48)	0.25
	Q4 (high)	126	2171.5	22	10.13	1.00		1.00	
						trend p=0.10		trend p=0.09	

Serum SOD activity (unit/ml)	Q1 (low)	156	2580.0	36	13.95	1.55 (0.90-2.66)	0.11	1.64 (0.92-2.91)	0.09
	Q2	115	1838.5	29	15.77	1.44 (0.83-2.50)	0.19	1.51 (0.86-2.67)	0.15
	Q3	116	1963.5	19	9.68	0.92 (0.50-1.71)	0.80	0.96 (0.51-1.81)	0.91
	Q4 (high)	120	2033.0	22	10.82	1.00		1.00	
						trend p=0.17		trend p=0.14	

Serum TBARS level (nmol/ml)	Q1 (low)	143	2411.5	26	10.78	0.88 (0.51-1.50)	0.63	0.85 (0.48-1.48)	0.56
	Q2	118	1951.0	24	12.30	1.05 (0.61-1.81)	0.86	1.06 (0.61-1.85)	0.85
	Q3	130	2145.0	27	12.59	0.87 (0.52-1.48)	0.61	0.92 (0.54-1.56)	0.76
	Q4 (high)	116	1908.0	29	15.20	1.00		1.00	
						trend p=0.87		trend p=0.87	

Hazard ratios (H.R.) and 95% confidence intervals (95%C.I.) were calculated using Cox proportional hazard model (StatView statistical package, Power Macintosh), after adjusting for sex and age (Model 1), or after adjusting for sex, age, habits of smoking status and alcohol consumption, and BMI (Model 2).

* : p < 0.05.

Mortality rates = [No. of deaths/ person years] × 1000

After adjusting for sex, age, BMI, smoking status and alcohol consumption (Model 2), the hazard ratios of subjects with the lowest blood and serum SOD activity levels and blood Zn/Cu ratios (Q1) to mortality from all causes were higher than for subjects with higher levels; the differences between the lowest (Q1) and highest (Q4) blood Zn/Cu ratios were statistically significant. The hazard ratios of serum TBARS levels to mortality from all causes were slightly lower for subjects with low serum TBARS levels, but the difference was not statistically significant, similar to the pattern seen above with hazard ratios to cancer mortality.

## DISCUSSION

The aim of this study was to assess whether there was a relationship between lower mortality rates from cancer (at all sites) or all causes among this population cohort and higher blood and serum SOD activity levels. Three isozymes of SOD, which dismutate superoxide anions and play an essential role in defense against oxidative stress in the human body, are known to exist: Cu, Zn-SOD derived from tissue cytosol of various tissues, Mn-SOD in mitochondrial matrix and extracellular SOD in extracellular fluids^11^^)^. Thus, serum and blood SOD activity levels are usually due to activity of Cu, Zn-SOD, released mainly from tissue cytosol and extracellular fluids, and from blood cells, respectively. Serum and blood SOD activity levels obtained in this study must be indicative of Cu, Zn-SOD activity. Serum and blood SOD activity levels observed in this study were similar to results reported elsewhere^32^^,^^33^^)^.

It has previously been shown that Cu, Zn-SOD detoxifies superoxide anions, which cause lipid peroxidation in various cells and tissue membranes and play a role in risk for various diseases, including cancer and cardiovascular diseases^3^^,^^7^^-^^10^^,^^34^^,^^35^^)^. There have been several case-control studies of correlation between cancer and Cu, Zn-SOD activity in serum and erythrocytes. In 2 studies, no correlation was found between serum Cu, Zn-SOD activity levels and several types of cancer^12^^,^^17^^)^. In 2 other studies, inverse correlations were found between SOD activity in erythrocytes and esophageal, gastric, colorectal, hepatic and pancreatic cancer^13^^,^^36^^)^. In a histochemical study, it was found that Cu, Zn-SOD protein and activity were significantly decreased in patients with colorectal carcinoma^37^^)^. It has also been reported that cellular proliferation and incidence of diploidy are high in cancer tissue that exhibits low SOD activity^38^^)^. Another study found that active superoxide production and low SOD activity may cause malignant cells to be highly sensitive to inhibition of SOD^39^^)^. In an experimental study, results suggested that SOD plays a role in dismutating reactive oxygen species that participate in the pathogenesis of various clinical disorders, and indicated that SOD functions as an antioxidant enzyme in cellular defense against oxidant-mediated tissue injury^40^^)^.

In the present population-based followed-up study, medical data obtained over the 18-year follow-up period showed an inverse correlation between cancer mortality and SOD activity in peripheral blood. In addition, the inverse correlation between SOD activity levels and cancer mortality rates was clearer for serum SOD activity than for blood SOD activity. These findings suggest that SOD activity, especially serum SOD activity, may be used as a biomarker in prevention of cancer mortality in the general population.

There was good correlation between blood levels of Zn and Cu obtained in this study by anodic stripping voltammetry and levels obtained by atomic absorption in other studies^41^^)^. Blood Zn and Cu levels we obtained were similar to results reported elsewhere^42^^,^^43^^)^. We observed relationships between blood Zn/Cu ratios and mortality rates from all causes.

It has been reported that Zn levels in plasma and erythrocytes are significantly lower in cancer patients than in controls, and that Cu levels in plasma and erythrocytes are significantly higher in cancer patients^44^^)^. We found that the hazard ratios of the lowest blood levels of Zn and Cu to cancer mortality rates were 1.30 (95% C.I., 0.56-3.02) and 0.97 (95% C.I., 0.40-2.35), compared to the highest levels, respectively, in the present cohort. It is well known that some trace metals (including Zn and Cu) act as cofactors of enzymes, in addition to other biological functions they may have^11^^,^^20^^)^. Zn and Cu have been shown to play a role in antioxidant activity via enzymatic oxidative reactions catalyzed by enzymes such as SOD and ceruloplasmin^3^^,^^11^^,^^19^^-^^22^^)^. Thus, the present findings also suggest that Zn is more effective than Cu as a cofactor in SOD activity and a protective factor in mortality from cancer and other diseases.

Moreover, TBARS is generally produced in part by the reaction of various unsaturated fatty acids contained in tissues with activated oxygen species such as hydroxyl radicals and superoxide anions^8^^-^^10^^)^. Superoxide anions are dismutated to oxygen and hydroperoxide by SOD, and this can reduce levels of lipid peroxides such as TBARS^3^^,^^11^^,^^20^^,^^35^^)^. Production of serum TBARS is known to be induced by unsaturated fatty acids^45^^,^^46^^)^ and to have a positive correlation with serum n-3 polyunsaturated fatty acid levels^47^^)^. However, it has been reported that serum TBARS levels are an unsuitable biomarker of lipid peroxide substances produced by lipid peroxidation^46^^,^^48^^)^. In the present study, although serum TBARS levels were similar to levels reported elsewhere^47^^,^^49^^,^^50^^)^, we did not obtain clear evidence (hazard ratios) of an inverse correlation between serum TBARS levels and mortality rates from cancer and all causes. It appears that serum TBARS levels are not reliable as a biomarker of risk for mortality from cancer and other diseases.

In conclusion, the results of the present follow-up study of rural Japanese inhabitants indicate that peripheral blood SOD activities, especially serum SOD activity, and blood Zn/Cu ratios may be useful as biomarkers of protective effects against mortality from cancer and other disorders, respectively. However, we did not observe a clear inverse correlation between low serum TBARS levels and mortality from cancer and/or all causes.
